# Novel 2D Dynamic Elasticity Maps for Inspection of Anisotropic Properties in Fused Deposition Modeling Objects

**DOI:** 10.3390/polym12091966

**Published:** 2020-08-30

**Authors:** Yuqi Jin, Teng Yang, Hyeonu Heo, Arkadii Krokhin, Sheldon Q. Shi, Narendra Dahotre, Tae-Youl Choi, Arup Neogi

**Affiliations:** 1 Department of Physics, University of North Texas, Denton, TX 76203, USA; yuqijin@my.unt.edu (Y.J.); hyeonu.heo@unt.edu (H.H.); arkadii.krokhin@unt.edu (A.K.); 2 Department of Mechanical and Energy Engineering, University of North Texas, Denton, TX 76207, USA; sheldon.shi@unt.edu (S.Q.S.); tae-youl.choi@unt.edu (T.-Y.C.); 3 Department of Materials Science and Engineering, University of North Texas, Denton, TX 76207, USA; tengyang@my.unt.edu (T.Y.); narendra.dahotre@unt.edu (N.D.); 4 Center for Agile and Adaptive Additive Manufacturing, University of North Texas, Denton, TX 76207, USA

**Keywords:** FDM, ultrasound elastography, 3D printing, additive manufacturing

## Abstract

In this study, a novel ultrasonic non-destructive and non-invasive elastography method was introduced and demonstrated to evaluate the mechanical properties of fused deposition modeling 3D printed objects using two-dimensional dynamical elasticity mapping. Based on the recently investigated dynamic bulk modulus and effective density imaging technique, an angle-dependent dynamic shear modulus measurement was performed to extract the dynamic Young’s modulus distribution of the FDM structures. The elastographic image analysis demonstrated the presence of anisotropic dynamic shear modulus and dynamic Young’s modulus existing in the fused deposition modeling 3D printed objects. The non-destructive method also differentiated samples with high contrast property zones from that of low contrast property regions. The angle-dependent elasticity contrast behavior from the ultrasonic method was compared with conventional and static tensile tests characterization. A good correlation between the nondestructive technique and the tensile test measurements was observed.

## 1. Introduction

3D printing technology, or additive manufacturing (AM) process, is one of the essential rapid prototyping techniques to develop three-dimensional complex shape parts. The layer-by-layer manufacturing of many AM processes enables the production of small volumes of customized objects, which are not achievable with traditional manufacturing technologies. The Fused Deposition Modeling (FDM), an extrusion-based AM process, is a fast, cost-effective, and user-friendly printing technique that is broadly involved in industries, academia, and even in the residential domain. The thermoplastic filament is melted in a heated nozzle and deposited on a platform bed layer by layer. The nozzle is connected to a three-axis motion system, which is controlled by G-code.

Most polymer 3D printers use either solid or liquid raw material to fabricate the desired geometry. Polylactic Acid (PLA) and Acrylonitrile butadiene styrene (ABS) are the most commonly used thermoplastic 3D printing materials for FDM [1]. As one of the most popular materials, ABS has many advantages, such as low cost, impact resistance, toughness, and higher glass transition temperature. 3D printing ABS [2] has been used for many applications in broad fields recently, such as fluid experimental setup [3] and dielectric devices [4,5]. Although raster width, layer thickness, and part orientation were shown to have a little impact on mechanical properties, the performance of 3D printed parts is significantly affected by printing parameters such as raster angle and the air gap between two adjacent deposited filaments within the same layer [3,4].

To evaluate the structural quality in terms of elasticity and plasticity of printed products, the mechanical tests are normally selected as the standard destructive testing methods, such as tensile and compression tests [6]. According to the ASTM standard D638 [7], specific dimensions of a specimen are recommended to characterize mechanical properties. The mechanical properties obtained in the narrow linear deformation range are strongly affected by the test stand and condition, e.g., the capacity and accuracy of loading cell and servo motor. Hence, an inadequate test stand is not able to measure properties correctly. In addition, it is well known that the infill path of the FDM products, auto generated by the slicing software based on the geometry of the model, has effect on the mechanical properties [2]. For instance, the 3D printed samples with different infill path have different tensile strength compared to the actual product without advanced editing. Moreover, the flaw in the infill paths design leads to the non-negligible variation in the local elasticity [8], thermomechanical residual stress [9], and even unexpected unfilled corners [10] in the printed parts. The existence of unfilled corners and other variations will greatly influence elasticity and plasticity in the FDM printed products. Hence, the characterized tensile strength by a tensile- or compression-test sample cannot accurately represent the actual mechanical property of the printed products with different geometries, or even printed under the same parameters.

Alternatively, cutting tensile samples from a 3D printed product can avoid the infill difference existing between the printed tensile sample and the printed products. However, cutting the sample from an already printed product can lead to heat generation that creates additional bonding and modifies the bounding between the infilled filaments. Thus, the internal residual stress due to heat generation would be partially released [11] and can affect the value of dynamic elasticity. Nanoindentation is another commonly applied elasticity testing technique, which is broadly applied in ceramic [12], alloys [13], and even biomass [14]. The typical tip size for the nanoindentation machine is about 1 µm [15], which is 300 times smaller than the typical diameter of FDM filament (300 µm). Hence, the nanoindentation test on the FDM product probes the mechanical property of an area either located on the infilled filament or at the boundary between the filaments instead of measuring the bulk scale elasticity of the products [16] as the layer structure is much larger than the tooltip.

Instead of the destructive test, a more reliable method for evaluating elasticity is a non-destructive ultrasound test [17]. The ultrasound test with transverse and longitudinal sound speed is a common non-destructive way to obtain elasticity of the material used for metals, alloys and other solid materials [18]. For the ultrasound test, the density and thickness values of the sample are needed to be pre-determined in the conventional speed of sound elasticity experiment in order to calculate the elasticity based on the speed of longitudinal and transversal sound wave [19]. In the measurement, the shear modulus of the material is obtained from the speed of the transverse wave and the density. The Young’s modulus and Poisson’s ratio can then be calculated from the shear modulus and the longitudinal speed of sound. However, in the inhomogeneous media, the ultrasonic measured dynamic elasticity is highly frequency dependent when the size of the ultrasound wavelength was close to the microstructure size in the media. The elasticity found from the ultrasound evaluation yields the information about the dynamic elasticity of the material and may not necessarily yield the same values of the magnitude as the static elastic modulus estimated from the tensile test of the inhomogeneous media [20,21], specifically, for composites material [22].

Many studies on the conventional ultrasonic evaluation used different operating frequency ranges of longitudinal and transversal sound waves, which introduced additional uncertainties in the evaluated dynamic modulus [23,24,25,26,27,28]. Moreover, the shear wave velocity evaluated shear modulus values are highly angle dependent in FDM printed layer objects, which has also barely been discussed in the existing literature. The elastography technique [29] is another conventional ultrasound technique that usually required external stress source [30] or periodical force (vibration) [31] to provide measurable elastic deformation using ultrasound. Thus, it is more suitable for soft material, such as ABS and PLA. Additionally, the listed existing ultrasound elasticity techniques are all contacting methods, which are highly limited to complex geometry and rough surface like the FDM products.

In this study, we introduce a novel technique to evaluate the dynamic elasticity in 2D contour maps of FDM products by a non-destructive and non-contacting ultrasonic method to overcome the limitations existing in the listed conventional method in the previous paragraphs. The recently investigated elastographic technique, Effective density and dynamic bulk modulus elastography (EBME) [32], can provide dynamic bulk modulus and effective density distribution of the tested objects by immersion ultrasound scan. To demonstrate EBME technique for inspection of anisotropic properties in FDM objects, two cubic shape samples with different printing conditions were used. And, the obtained results were validated through the conventional tensile test using the printed tensile samples.

In the Section 2 and Section 3, the material preparation and EBME theory are described in detail. The effective density and dynamic bulk modulus disributions in the 2 printed blocks were compared to a reference ABS block made by conventional molding process in the Section 4.1. The properties zones in both samples were clearly illustrated in the elastography comparing with the reference block. Furthermore, for anisotropic FDM parts [16,33], angle-dependent shear wave test conducted angle-dependent dynamic Young’s modulus and dynamic shear modulus maps, which has never been studied and demonstrated in the existing literature in the Section 4.3 and Section 4.4. To verify the angle-dependent anisotropic elasticity behaviors estimated from Ultrasonic EBME test, we conducted tensile tests on the infill angle-dependent tensile samples to obtain the static anisotropic Young’s modulus in the Section 4.5. The results showed the angle-dependent static Young’s modulus behaviors from the conventional tensile test was a good agreement with dynamic Young’s modulus estimated from ultrasound measurement. The determined information is invaluable for inspecting the relative quality of the FDM products and offers a non-invasive alternative solution for the quality monitoring of FDM products.

## 2. Materials and Methods

### 2.1. FDM Printed Samples

In this study, both ultrasonic testing and tensile test samples were printed using PolyPrinter 229 single extruder FDM printer (Polyprinter, Midlothian, TX, USA) with a close shell using ABS (Acrylonitrile Butadiene Styrene) filament from Hatchbox (Pomona, CA, USA). The designed 3D models were converted to G-Code through KISSlicer software (www.kisslicer.com). The extruder temperature was set at 260 °C for the first layer and at 255 °C for the remaining layers. The heated printing substrate was covered by a smooth tape and the temperature was maintained at 110 °C. The value of the nozzle size in the slicing software was set from the values of the height of the printing layer and the actual nozzle size of the extruder. The shape of the cross-section of the extruded filament can be considered as circular [34]. The number of layers of the skin, top and bottom, were set to 2. The printed samples were both printed at a 100% infill setting condition.

**Figure 1:**
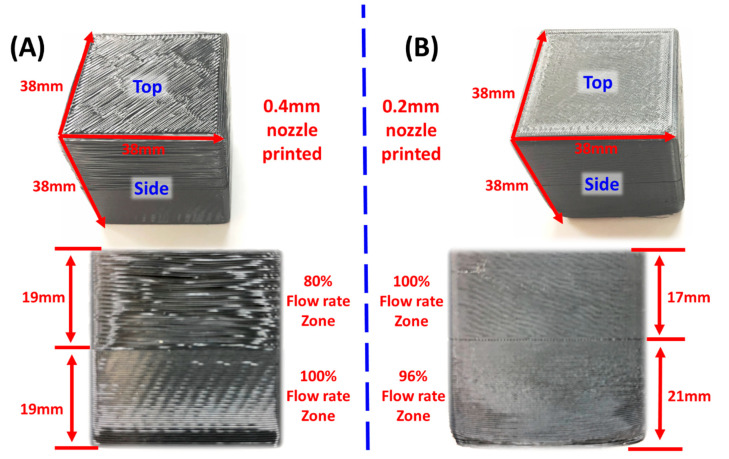
FDM printed ABS blocks for elastography testing of size 38 mm *×* 38 mm *×* 38 mm. (**A**) Sample 1 was printed using a 0.4 mm nozzle under a 100% flow rate and an 80% flow rate setting. The height of both regions was 19 mm. (**B**) Sample 2 was printed using a 0.2 mm nozzle under a 96% flow rate region with a height of 21 mm and a 100% flow rate zone had the height 17 mm.

In the present work, two 38 mm wide cubes were printed for elastography testing. As shown in Figure 1, sample 1 was printed by a 0.4 mm nozzle with half the height at 100% flow rate and the other half at 80% flow rate and exhibit a large contrast in physical properties. Sample 2 was printed by a 0.2 mm nozzle with 21 mm height at 96% flow rate and the rest 17 mm height at 100% flow rate. The two regions have a very limited variety of physical characteristics between the two zones.

### 2.2. Tensile Test Samples with Various Infill Angles

**Figure 2:**
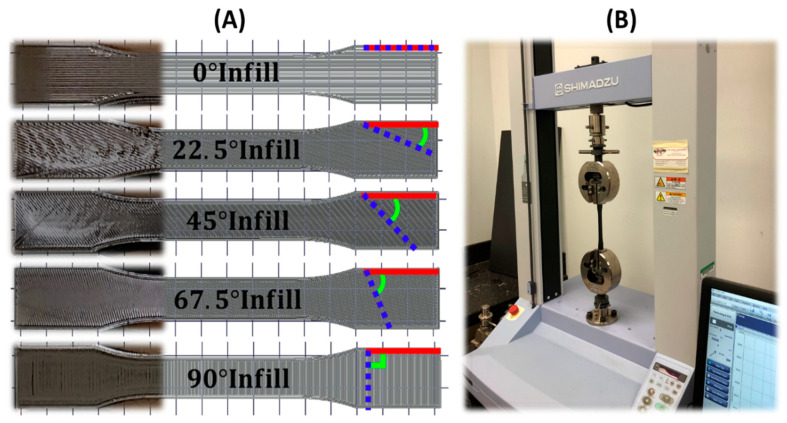
(**A**) Designed and printed ABS tensile samples with 0°, 22.5°, 45°, 67.5°, and 90° infilled angles illustrated by photographs and slicing path together. The tensile force on all the samples is along the horizontal direction. (**B**) Photograph of the involved 10 KN tensile test machine with a printed tensile sample attached.

The ABS samples for the tensile test was printed with five different infill angles, 0°, 22.5°, 45°, 67.5°, and 90°, and two nozzle sizes at 100% infill setting (Figure 2A). We prepared three identical samples for each combination. A total of 30 samples and some extra pieces for calibration of the machine was tested. The printed tensile samples and tensile test procedure (Figure 2B) were based on the ASTM D638 standard [7]. SHIMADZU AGS-X universal testing machine with a 10 KN calibrated load cell (Kyoto, Japan) was used for the tensile test. The calibration and the zero set of the load cell were performed before testing on all the samples. The testing motion was set to stop-at-break with the maximum load at 10 KN and the maximum displacement at 20 mm. The measured displacement and the applied load through the tensile test were converted to the stress and the strain based on the given cross-section area (13 mm × 3.2 mm) and length (57 mm) of the specimen. Then, the static Young’s modulus was estimated from the ratio of the stress to the strain.

### 2.3. Dynamic Bulk Modulus and Effective Density Elastography (EBME)

**Figure 3:**
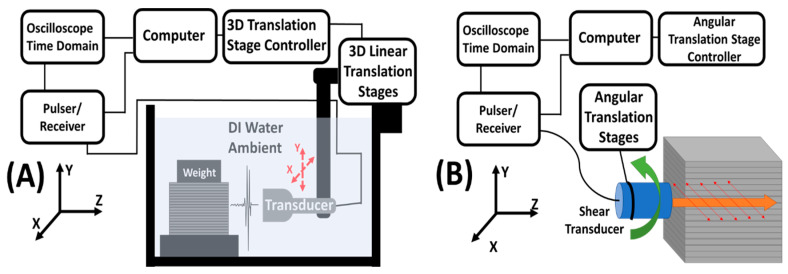
(**A**) Experimental setup of the immersion elastographic (EBME) raster scan. (**B**) Experimental setup of the angle-dependent shear wave velocity test. The angle indicated the slant between the infilled planes (along the X-axis) on the samples and the plane shear wave propagating. The shear (transverse) wave propagating along the Z-direction and oscillating in the XY plane. 0° indicated the shear wave oscillated along the X-axis and 90° referred the shear wave oscillated along the Y-axis.

The EBME tests were performed in a homemade acrylic water tank (500 mm × 500 mm × 500 mm) infilled with DI water. The ultrasound transducer and tested samples were immersed underwater as shown in Figure 3A. The scanned area on both the 3D printed blocks and the reference block were 20 mm × 20 mm with a 1 mm step interval. In the scanned area, the temporal data of each location (441 points) was acquired and recorded in a square matrix. An Olympus V316-N-SU (Olympus-IMS, Waltham, MA, USA) 0.125-inch diameter 20 MHz unfocused emersion pencil style transducer was used to excite a broadband pulse from 10 to 35 MHz with a repetition rate of 200. The raster-scanned reflection was collected from 441 locations during the raster scan. The entire raster scanning process including automated movement sequences of a transducer and data acquisition was programmed using MATLAB^®^ script. The transducer was connected to the 3 axes translation stages, LC Series Linear Stages of Newmark Systems, Inc., and controlled by the 3 axes motion controller. A JSR Ultrasonic DPR 500 Pulse/Receiver (Imaginant, Inc., Pittsford, NY, USA) internally operated the pulse source and time trigger, and the data was collected by a Tektronix MDO 3024b (Tektronics Inc., Beaverton, OR, USA). The data acquisition rate was 512 signals per 20 s. At each measured location in the raster scan, the 512 signals were averaged to 1 recorded waveform in the oscilloscope.

### 2.4. Speed of Sound Measurement of Shear Wave

The angle-dependent shear wave velocity test was performed using an Olympus V222-BC-RM 20 MHz (Olympus-IMS, Waltham, MA, USA) normal incidence transverse transducers in the 3D printed block and the reference block as shows in Figure 3B. The motorized continuous rotation stage was used to control the angles between the horizontal building plane and the direction of the oscillation of the shear wave. The stage was driven by Thorlabs DC motor (Thorlabs Inc., Newton, NJ, USA). In this study, five different angles, such as 0°, 22.5°, 45°, 67.5°, and 90°, were chosen to show the angle-dependency of the shear wave.

## 3. Theory

The dynamic bulk modulus and effective density can be calculated by the measured acoustic impedance and sound velocity of the tested sample [31]. The acoustic impedance ratio between the sample and known ambient reference fluid (DI-water) is given by:

(1) $$\frac{Z_{1}}{Z_{0}}=\frac{-1-\alpha -\sqrt{4\alpha +1}}{\alpha -2},$$

where $\alpha =\;\frac{P_{1}}{\;P_{e}-sgn\left(Z_{1}-Z_{0}\right)\left|P_{0}\right|}$ was measured from the experimental measurement. $P_{e}$, $P_{1}$ and $P_{2}$ are the amplitude of the transducer emitted pulse, first refection, and second refection, respectively. $Z_{0}$ is the known acoustic impedance of ambient material, ambient water. $Z_{1}$ is the target impedance of tested samples. Subscript 0 and 1 referred to the ambient material (DI water) and the printed samples.

The dynamic bulk modulus is written as:

(2) $$K_{dyn}=c_{L}Z_{0}\frac{-1-\alpha -\sqrt{4\alpha +1}}{\alpha -2}.$$

The effective density can be expressed as:

(3) $$\rho_{eff}=\frac{Z_{0}}{c_{L}}\frac{-1-\alpha -\sqrt{4\alpha +1}}{\alpha -2},$$

where $c_{L}$ is the speed of the longitudinal wave in the time domain of the two recorded echoes?

The angle-dependent dynamic shear modulus can be obtained by the effective density and the speed of sound of the transverse wave:

(4) $$G_{dyn}\left(\theta \right)=\rho_{eff}c_{T}{\left(\theta \right)}^{2},$$

where $c_{T}\left(\theta \right)$ is the angle-dependent speed of the transverse sound wave measured by monostatic time of flight method, and θ is the angle is defined as the inclined angles between the printed layers and the shear wave oscillation plane.

The angle-dependent dynamic Young’s modulus can be calculated as follows:

(5) $$E_{dyn}\left(\theta \right)\;=\frac{9K_{dyn}\;G_{dyn}\left(\theta \right)\;}{3K_{dyn}\;+\;G_{dyn}\left(\theta \right)}.$$

## 4. Results

### 4.1. Dynamic Bulk Modulus Elastographies and Effective Density Maps

**Figure 4:**
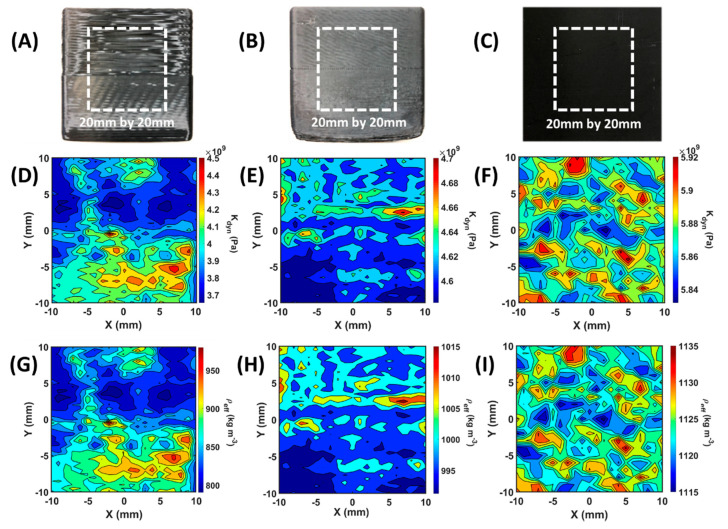
Dynamic bulk modulus imaging and effective density maps from immersion ultrasonic test based on the EBME method. (**A**–**C**) The photograph of the side view of ABS samples, such as printed by a larger nozzle, sample 1, (**A**), printed by a finer nozzle, sample 2, (**B**), and the reference block made by the injection molding process (**C**), respectively. The reference molded sample does not have layer structure. (**D**–**F**) Dynamic bulk modulus imaging, calculated by Equation (2), of sample 1 (**D**), sample 2 (**E**), and the reference molded sample (**F**), respectively. (**G**–**I**) Effective density maps, obtained from Equation (3), of sample 1 (**G**), sample 2 (**H**), and the reference molded sample (**I**).

In Figure 4, the dynamic bulk modulus elastographies and the effective density maps of two printed samples and the reference molded sample were calculated by Equations (2) and (3), respectively. Parameters in Equations (2) and (3) were measured through the longitudinal wave immersion test with the raster scan on each sample. The scanned areas were all 20 mm by 20 mm with a 1 mm interval on both the X- and Y-axis as the white dash squares indicated in Figure 4A–C. The scanned areas were all aligned to the center of the side surfaces of the samples. For instance, in the scanned area of sample 1, both maps, Figure 4D,G, showed that the interface between relatively higher (100% flow rate) and lower regions (80% flow rate) of properties is located at the center. For sample 2, the interface is not at the center, but 2 mm above the centerline. The results of elastography and density map, Figure 4E,H, showed that the interface between two regions having different properties is located at the expected position, 2 mm on the Y-axis.

The boundary between the zones was clearer in sample 2 rather than sample 1 due to better stability during fabrication by a finer nozzle. In the injection molded reference ABS block results, the small variation in dynamic bulk modulus and effective density were randomly distributed due to the internal residual stress from the non-uniform cooling process. The average dynamic bulk modulus and effective density of the molded sample was 5.88 GPa and 1125 kg m^−3^. The variation in the scanned dynamic bulk modulus and the effective density of the molded sample were about 1.5% and 1.8%, respectively. In the printed sample 1 and 2, the property regions were artificially designed and fabricated. In sample 1, the average dynamic bulk modulus values were 4.12 GPa in the 100% flow rate region and 3.82 GPa in the 80% flow rate region with 15% and 10% variation, respectively, as shown in Figure 4D,G. The average effective density values from the two regions were 892 kg m^−3^ and 825 kg m^−3^ with 17% and 11% variation, respectively. From Figure 4B,E,H, the finer nozzle printed sample 2 provided much more uniform printing quality in terms of variation in the properties on the sample. The average dynamic bulk modulus values were 4.63 GPa with 1.8% variation in the upper side printed with 100% flow rate region and 4.59 GPa in the 96% flow rate region with a 1.5% variation. The average effective density values of the 100% flow rate were 1003 kg m^−3^ with 2.0% variation and 994 kg m^−3^ with 1.9 % variation in the 96% flow rate zone. The elasticity variation between the two zones in sample 2 was about 23%. The elasticity of finer printed sample with 100% infill setting yielded an elasticity value which was 80% of the conventionally molded reference block. The bulk properties in FDM 3D printed products were far from the elastic properties and density of a conventional molded block due to unavoidable porosity (air package).

### 4.2. Angle-Dependent Shear Wave Velocity

The dynamic bulk modulus elastography and the effective density map through the longitudinal wave test can provide the angle-independent properties of samples, but the directional properties cannot be estimated. The properties of FDM products, however, actually have directionality due to the layers in the structures. To inspect or monitor a printed sample precisely, the method that can provide angle-dependent properties, e.g., Young’s modulus and shear modulus, is necessary. In this section, the shear wave test with varying angles was conducted at the same frequency as the longitudinal test to properly estimate the anisotropy in the FDM products. 

**Figure 5:**
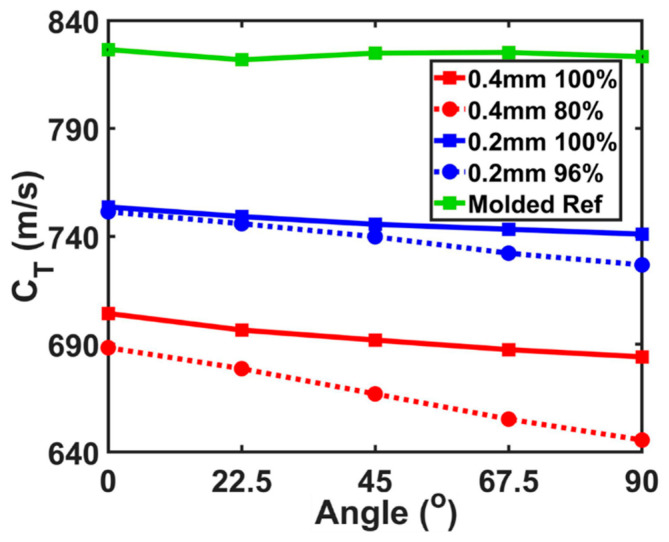
Angle-dependent speed of shear wave measured at 0°, 22.5°, 45°, 67.5°, and 90°. Green line represents the results from the reference block. Blue solid (dot) line is the measurement on the sample 2 at 100% (96%) flow rate printed region. Red solid (dot) line is the measurement on the sample 1 at 100% (80%) flow rate printed region.

In Figure 5, the angle-dependent shear wave velocity, $c_{T}$, results of each property zone from the two printed samples were illustrated along with the measurement on the reference molded sample. The angle is defined as the inclined angles between the printed layers and the plane shear wave propagating. The green line shows the speed of the shear wave in the reference molded sample, which was angle-independent because of the lack of layer structure. On the other hand, in the FDM printed sample 1 and 2, the shear wave was oscillating and propagating along the layers at 0° and across the layers at 90° at the measured location. The angle-dependent shear wave velocity showed a decreasing behavior along the increasing of the angle. In the 100% flow rate zone of the 0.4 mm nozzle printed sample 1 (red solid line), the shear wave velocity decreased 3.1% from 0° to 90°. In the 80% flow rate zone (red dot line), a dramatic drop in the velocity was observed at about 6.7%. In the 100% flow rate region of the 0.2 mm nozzle printed sample 2 (blue solid line), the shear wave velocity decreased 1.7% from 0° to 90°. In the 96% flow rate zone (blue dot line), the larger drop shown in the shear wave velocity was determined as 3.4%.

### 4.3. Angle-Dependent Dynamic Shear Modulus

**Figure 6:**
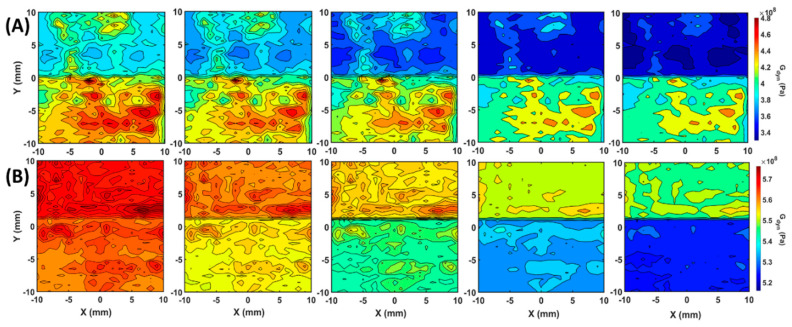
Angle-depedendent dynamic shear modulus distribution maps calculated by Equation (4) with $\rho_{eff}$ and $C_{T}$. Row (**A**) and (**B**), from left to right, the dynamic shear modulus maps at 0°, 22.5°, 45°, 67.5°, and 90° of sample 1 and sample 2, respectively.

With the evaluated speed of the shear wave and the effective density from the immersion longitudinal wave EBME scan, the directional dynamic shear modulus values were calculated by Equation (4). As shown in Figure 6, row (A) and (B) illustrate the directional shear modulus maps of samples 1 and 2 which described the transversal elasticity at 0°, 22.5°, 45°, 67.5°, and 90°. The color scales of subfigures in rows (A) and (B) were normalized to the same ranges for a better view on angle-dependent behaviors. Both sample 1 and 2 showed that the contrast of the dynamic shear modulus between the different property zones became larger proportional to the angle between the filled planes and the measured orientation. As expected, the dynamic shear modulus values at 0° degree were determined as the maximum because the local elasticity along the printed planes was higher than the local elasticity values crossing the constructed planes. The average shear modulus contrasts between the two regions on sample 1 were 43 MPa at 0°, 48 MPa at 22.5°, 54 MPa at 45°, 61 MPa at 67.5°, and 66 MPa at 90°. The average absolute dynamic shear modulus values of the two regions underwent a significant reduction from 388 MPa and 431 MPa at 0° to 338 MPa and 404 MPa at 90°. In the finer constructed sample 2, the average angle-dependent dynamic shear modulus values in the two property zones decreased from 575 and 568 MPa at 0° to 548 and 521 MPa at 90°. The average shear modulus contrasts between the two regions on sample 1 were 7 MPa at 0°, 10 MPa at 22.5°, 17 MPa at 45°, 20 MPa at 67.5°, and 27 MPa at 90°. The angle-dependent elasticity indicates that the measured values were varying from the horizontal (0°) to vertical (90°) by a 22.5° interval. FDM printed samples are in the form of the layered structure. The 0° elasticity was described along the planes and 90° elasticity referred to across the planes, which were expectedly different due to the filament binding in planes and across planes.

### 4.4. Angle-Dependent Dynamic Young’s Modulus

**Figure 7:**
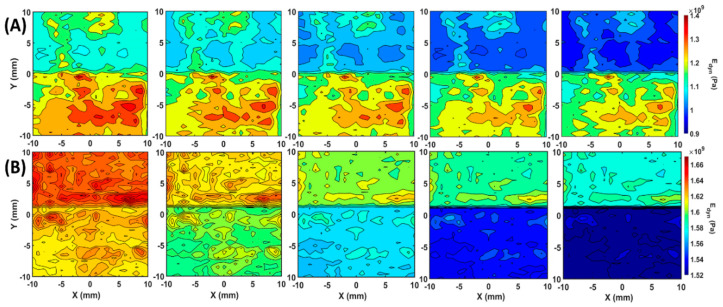
Angle-depedendent dynamic Young’s modulus distribution maps calculated by the $K_{dyn}$ and $G_{dyn}$ by Equation (5). Row (**A**), from left to right, the dynamic shear modulus maps at 0°, 22.5°, 45°, 67.5° and 90° of sample 1. Row (**B**), from left to right, the dynamic shear modulus maps at 0°, 22.5°, 45°, 67.5° and 90° of sample 2.

From the dynamic bulk modulus and dynamic shear modulus, the dynamic Young’s modulus distribution can be obtained on the scanned area using EBME. In Figure 7, row (A) illustrated the angle-dependent dynamic Young’s modulus maps of sample 1 at 0°, 22.5°, 45°, 67.5°, and 90°, while row (B) indicated the angle-dependent dynamic Young’s modulus maps of sample 2 along with the angles. In general, the dynamic Young’s modulus maps in Figure 7 showed a more significant contrast between the property zones than the dynamic shear modulus in Figure 6. In the dynamic Young’s modulus maps of sample 1, the average dynamic modulus in the upper zone was 1134 MPa at 0° and 962 MPa at 90°. In the lower half of the images with enhanced material property, the average dynamic bulk modulus values were 1252 MPa at 0° and 1174 MPa at 90°. In sample 2, as row (B) showed, the average dynamic modulus was 1655 MPa and 1632 MPa at 0°, 1574 MPa, and 1525 at 90°. The detail dynamic modulus values of each regions of both blocks were listed in the Table A1 in Appendix A.

### 4.5. Infill Angle-Dependent Static Young’s Modulus from Tensile Test

**Figure 8:**
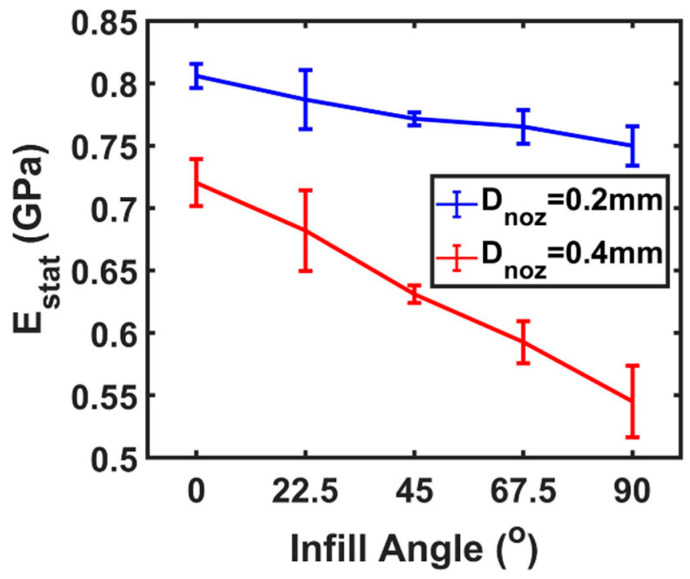
The static Young’s modulus versus the infill angle measured by the tensile test of printed ABS tensile specimens with 100% infill. Blue (red) line indicates the nozzle size of the extruder, D_noz_, i.e., the specimen is printed by a 0.2 mm (0.4 mm) nozzle. Each data point is the averaged value and the error bars represent the standard deviation.

To verify the observed angle-dependent dynamic Young’s modulus behavior existing in the FDM printed samples, we conducted infilled angle-dependent tensile tests for determining the static modulus as shown in Figure 8. For remaining the stable quality of the printed tensile samples, only a 100% flow rate setting was applied to avoid wrapping problems on the tensile samples. The trend of the tested static Young’s modulus of printed samples using both nozzles, 0.2 and 0.4 mm, followed infilled angle dependence. The highest modulus obtained at 0°, indicating the infill direction matched with the tensile stress direction. When the angle between the infill direction and tensile stress direction became more substantial, the observed Young’s modulus was minimal. Furthermore, similar to the dynamic Young’s modulus maps illustrated, the larger printing nozzle provides more dramatic angle dependence in the static modulus. The absolute values in the static Young’s modulus were about half as the dynamic Young’s modulus, which was not due to the dispersion of sound existing in viscoelastic media [35], but because of the smaller oscillating area in the ultrasound test at each scanned location. In the high operating frequency, the micro-meter scale wavelength propagated the energy more along the infilled filaments with less effect from the air package inside of printed samples. However, in the static tensile test, the weight of bulk scale factors take account into the mechanical response to the tensile stress such as unfilled slicing path, residual stress, air packages, and the bonding between the infilled filament.

## 5. Discussion

In our previous work [36], we have demonstrated the noninvasive method to evaluate the FDM printing quality by low frequency tested effective density maps (EBME). The effective density could be converted to a volumetric fraction of internal air packages. The effective density evaluation was able to determine the unexpected variation in flow rate caused by changes in tool conditions such as nozzle, extruding gear, and heater. In this study, the EBME method was evolved to provide more precise 2D elasticity maps to inspect the FDM printed objects. The elasticity maps were able to present non-directional elasticity, dynamic bulk modulus, and anisotropic elasticity such as dynamic shear modulus and dynamic Young’s modulus. In the future, the resolution of the dynamic elasticity could be further improved by an acoustic lens to reduce the beam width [37].

From the presented elasticity maps, the non-directional dynamic bulk modulus maps presented high-frequency incompressibility of the FDM printed samples, which does not show as clearly as the contrast found in dynamics shear modulus and dynamic Young’s modulus maps. As more commonly used elasticity, shear modulus and Young’s modulus in FDM-printed objects are highly directional dependents due to the layers structure’s anisotropy. In conventional methods, as we also demonstrated, the tensile test for determining angle-dependent elasticity is destructive and time-consuming. The specific designed and sliced samples needed to be fabricated and tested, which is suitable for manufacturing studies instead of practical applications. In the real FDM products, the parts can barely be tested by a destructive method. Moreover, the uniquely designed tensile test samples could not fully predict the elasticity in a real product with different geometry due to slicing processing and accuracy of the printer motors. The noninvasive dynamic elasticity elastography technique demonstrated in this study provides a promising alternative solution for evaluating the anisotropic elasticity distribution in the FDM products and overcoming the limitations of conventional techniques.

## 6. Conclusions

In this study, a novel ultrasonic evaluation technique, based on recently developed dynamic modulus and effective density maps, was designed for additive manufacturing to image the directional dynamic shear modulus and dynamic Young’s modulus distributions in FDM printed objects. The anisotropy in the elastic properties of 3D printed material was mapped using a nondestructive ultrasound scan. The 3D printed samples were artificially designed with different characteristics regions having both high contrast and low contrast by adjusting the flow rate within the setting of the printing process. The dynamic shear modulus and dynamic Young’s modulus maps provided outstanding imaging and evaluation of the various property zones. Moreover, the angle-dependent anisotropic elasticity behavior was provided by the ultrasound scan as well, which was verified with conventional tensile tests.

## Appendix A

**Table A1:** Summarized angle-dependent dynamic Young’s modulus values in MPa.

	E_dyn_ @ 0°	E_dyn_ @ 22.5°	E_dyn_ @ 45°	E_dyn_ @ 67.5°	E_dyn_ @ 90°
Sample 1 100%	1252	1225	1203	1192	1174
Sample 1 80%	1134	1082	1054	1015	962
Sample 2 100%	1655	1621	1602	1592	1574
Sample 2 96%	1632	1606	1576	1555	1525
